# Characterizing Artificial Intelligence Psychosis in a Large Medical Setting

**DOI:** 10.1001/jamapsychiatry.2026.2923

**Published:** 2026-10-07

**Authors:** Zachary Bergson, Sarah G. Vassall, Adam Wright, Allison B. McCoy, Katherine M. Schafer, Margaret C. Achee, Julia M. Sheffield

**Affiliations:** 1 Department of Psychiatry and Behavioral Sciences, Vanderbilt University Medical Center, Nashville, Tennessee; 2 Department of Biomedical Informatics, Vanderbilt University Medical Center, Nashville, Tennessee

## Abstract

This cohort study estimates the prevalence of AI psychosis, characterizes affected patients, and defines the role of conversational AI in documented psychotic episodes.

Conversational artificial intelligence (AI) chatbots are increasingly linked to emerging or worsening psychotic symptoms, a phenomenon termed *AI psychosis*.^1^ Existing evidence, however, is limited primarily to case reports, viewpoints, and simulated experiments, with one published analysis of its occurrence in Denmark.^2^ While these works offer critical starting points, additional empirical data are needed to elucidate the factors characteristic of those who develop AI psychosis. Thus, we conducted a retrospective electronic health record (EHR) review at a large academic medical center to estimate prevalence, characterize affected patients, and describe the role of conversational AI in psychotic symptoms.

## Methods

This cohort study followed the STROBE reporting guideline and was approved by the Vanderbilt University Medical Center (VUMC) IRB with a waiver of informed consent for secondary analysis of already collected data. Using Epic Clarity, we extracted progress notes dated December 1, 2022, to April 15, 2026, from patients who received behavioral health care and whose records contained AI-related keywords (eFigure 1 in Supplement 1). Only patients with a documented psychotic-disorder diagnosis were retained. Two raters (Z.B., S.G.V.) manually screened notes, discarded incidental matches, and classified each patient’s AI interaction into 1 of 3 categories: neutral (eg, using ChatGPT to search for recipes), AI psychosis (psychosis-worsening chatbot use), or AI-related psychotic content (AI incorporated into a delusion without documented chatbot use; included to capture the range of AI-related phenomenology in the EHR while preserving the AI psychosis category for patients with clear use of a conversational AI product). Within the AI psychosis group, Z.B. and M.C.A. assigned interactions to 1 of 4 a priori theoretical categories describing how chatbot use interacts with psychosis^7^: catalyst (precipitates new symptoms in previously healthy individuals), amplifier (worsens preexisting symptoms by validating distorted ideas), coauthor (develops harmful narratives through iterative dialogue), or object (AI becomes the focus of delusional beliefs). Finally, across all patients, medical record review was conducted (Z.B., J.M.S., and M.C.A.) to determine whether the documented AI interaction was part of the patient’s first psychotic episode (index episode), defined as the AI interaction being documented by their treatment team during their first psychiatric presentation for psychotic symptoms.

Kruskal-Wallis, χ^2^, and Fisher exact tests were used to compare variables across groups. A 2-sided *P* < .05 was considered significant.

## Results

Among 578 058 records (215 712 unique patients), 187 encounters from 73 unique patients (25 [34.2%] female and 48 [65.8%] male; median [range] age, 32 [17-81] years) met criteria after algorithmic and manual screening. Twenty-eight patients (0.013% of those receiving mental health care) were classified into the AI psychosis category; 17 were classified as neutral; and 28 were classified into the AI-related psychotic content category. Percent agreement among raters was 86.1%, with J.M.S. serving as tiebreaker.

The AI psychosis cohort was primarily male (19 [67.9%]) and White (21 [75.0%]). Unspecified psychosis was the most common diagnosis (8 [28.6%]) (Table). ChatGPT was the documented AI product in 15 cases (53.6%), and 24 interactions (85.7%) were documented after the May 2024 release of GPT-4o.

**Table.** Demographic and Clinical Characteristics

Characteristic^a^	No. (%)			*χ^2^* (*df*)	*P* value
	AI psychosis (n = 28)	Neutral interaction (n = 17)	AI-related psychotic content (n = 28)		
Age, median (range), y	27.5 (17.0-66.0)	29.0 (18.0-81.0)	36.5 (18.0-70.0)	9.29 (2)	.009
Sex					
Female	9 (32.1)	2 (11.8)	14 (50.0)	6.95 (2)	.03
Male	19 (67.9)	15 (88.2)	14 (50.0)		
Race and ethnicity^b^					
African American or Black	4 (14.3)	3 (17.6)	4 (14.3)	4.73 (8)	.97^c^
Asian	2 (7.1)	0	1 (3.6)		
Hispanic	1 (3.6)	0	0		
Middle Eastern or North African	0	0	1 (3.6)		
White	21 (75.0)	14 (82.4)	22 (78.6)		
Prior psychiatric hospitalizations	16 (57.1)	14 (82.4)	22 (78.6)	4.47 (2)	.12
History of psychotropic medications	25 (89.3)	16 (94.1)	24 (85.7)	0.77 (2)	.68
History of outpatient treatment	24 (85.7)	16 (94.1)	22 (78.6)	2.02 (2)	.36
History of suicide attempts	5 (17.9)	7 (41.2)	9 (32.1)	3.06 (2)	.22
First psychotic episode	17 (60.7)	3 (17.6)	8 (28.5)	10.1 (2)	.006
Primary diagnosis					
Bipolar disorder with psychotic features	7 (25.0)	3 (17.6)	3 (10.7)	29.1 (16)	.001^a^
Brief psychotic disorder	3 (10.7)	0	1 (3.6)		
Delusional disorder	0	0	1 (3.6)		
MDD with psychotic features	1 (3.6)	0	0		
Schizoaffective disorder	0	3 (17.6)	4 (14.3)		
Schizophrenia	7 (25.0)	8 (47.1)	5 (17.9)		
Schizophreniform disorder	1 (3.6)	2 (11.8)	0		
Substance-induced psychosis	1 (3.6)	1 (5.9)	0		
Unspecified psychosis	8 (28.6)	0	14 (50.0)		
AI psychosis category					
Amplifier	18 (64.3)	NA	NA	NA	NA
Object	6 (21.4)	NA	NA		
Catalyst	3 (10.7)	NA	NA		
Coauthor	0	NA	NA		
None	1 (3.6)	NA	NA		

Abbreviations: AI, artificial intelligence; *df*, degrees of freedom; MDD, major depressive disorder; NA, not applicable.

^a^ Demographic and clinical features were extracted from the electronic health record.

^b^ Race and ethnicity were included in this analysis to fully characterize the demographics of the sample.

^c^ *P* values for the primary diagnosis and race between-group comparisons were generated using Fisher exact tests (Freeman-Halton extension) with 100 000 Monte Carlo simulations, owing to sparse cells.

Patients with AI psychosis did not significantly differ from those with neutral interactions or AI-related psychotic content on any clinical history variables, such as history of psychiatric hospitalization, psychotropic prescription, or suicide attempts (Table). Seventeen patients in the AI psychosis group (60.7%) were experiencing a first psychotic episode, which was significantly more than the neutral (3 [17.6%]; *P* = .006) or AI-related content (8 [28.5%]; *P* = .03) groups (Figure). Patients with AI psychosis were significantly younger than the AI-related psychotic content group (median [range] age, 27.5 [17.0-66.0] vs 36.5 [18.0-70.0] years; *W* = 3.931; *P* = .02) and more likely to have unspecified psychosis than the neutral group (8 [28.6%] vs 0; Holm-adjusted *P* = .03).

**Figure.**
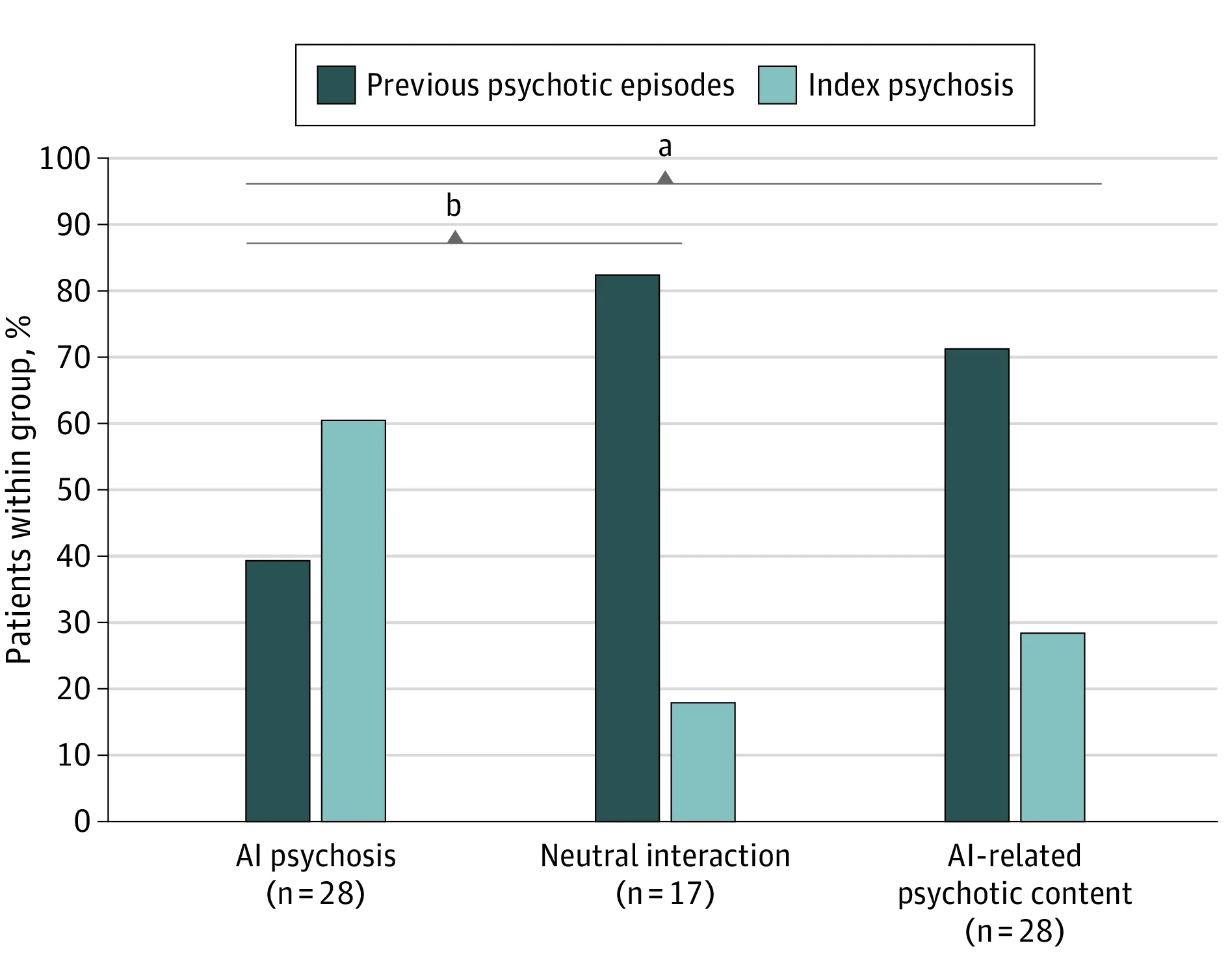
Bar Graph of Percentage of Patients Experiencing First Episode (Index) Psychosis Alongside a Documented Interaction With Artificial Intelligence (AI) The AI psychosis group had significantly more index psychosis cases than the neutral interaction group and the AI-related psychotic content group. ^a^*P* = .03 for AI psychosis vs AI-related psychotic content. ^b^*P* = .006 for AI psychosis vs neutral interaction.

Among AI psychosis cases, amplifier was the most common AI psychosis type (18 [64.3%]), followed by object (6 [21.4%]) and catalyst (3 [10.7%]) (Table). Percent agreement was 83.3% (J.M.S. as tiebreaker).

## Discussion

In this systematic analysis of AI psychosis, documented in the EHR of a large US medical setting, psychosis-worsening interactions with conversational AI were most common among patients experiencing their first episode of psychosis. Exploratory analysis of theoretical typologies^7^ has suggested conversational AI may amplify emerging or pre-existing symptoms. Our findings suggest that the early phase of psychotic illness may confer particular vulnerability to symptom-worsening AI interactions.

Our AI psychosis prevalence estimate of 0.013%, which is conditional on a patient receiving a psychosis diagnosis in the EHR, converges with evidence provided by OpenAI, which acknowledged “0.07% of users active in a given week and 0.01% of messages indicate possible signs of mental health emergencies related to psychosis or mania.”^8^ The high number of cases after the release of GPT-4o coincides with documented sycophancy in that model,^9^ though our design cannot establish causation. Recent simulation studies have identified GPT-4o as performing particularly poorly during extended conversations, and the more recent OpenAI model GPT-5.2 Instant as being more likely to challenge delusional premises.^10^

Limitations include ascertainment bias, single-site design, and a rating system that is not clinically validated. Prospective studies incorporating chat logs are needed to clarify mechanisms. Routine assessment of AI use during psychiatric encounters appears warranted.

## Notes

Supplement 1. **eFigure.** Electronic health record screening workflow

Supplement 2.
Data Sharing Statement
