# Supplementary material for: Characterizing Artificial Intelligence Psychosis in a Large Medical Setting
Source: JAMA Psychiatry. 2026 Oct 7:e262923. Online ahead of print. doi: 10.1001/jamapsychiatry.2026.2923 (PMC13647186; doi:10.1001/jamapsychiatry.2026.2923)
Supplement: Supplementary file 1 [file jamapsychiatry-e262923-s001.pdf]

Supplemental Online Content

Bergson Z, Vassall SG, Wright A, et al. Characterizing artificial intelligence psychosis in a large medical setting. *JAMA Psych*. Published online October 7, 2026. doi:10.1001/jamapsychiatry.2026.2923

eFigure. Electronic health record screening workflow

This supplemental material has been provided by the authors to give readers additional information about their work.

© 2026 Bergson Z et al. JAMA Psychiatry.

eFigure 1. Electronic health record screening workflow

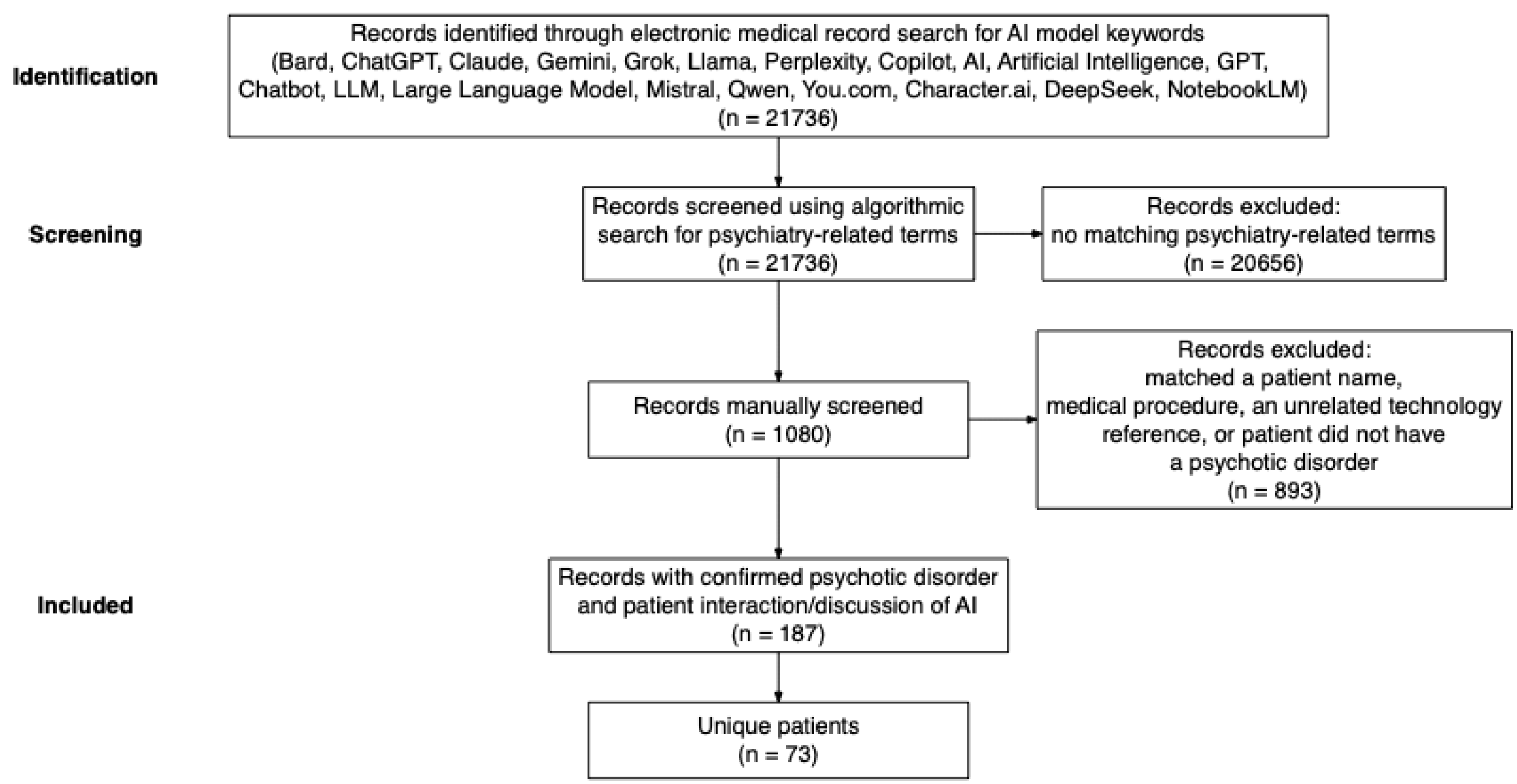

© 2026 Bergson Z et al. JAMA Psychiatry.
