# Supplementary material for: Characterizing Artificial Intelligence Psychosis in a Large Medical Setting
Source: JAMA Psychiatry. 2026 Oct 7:e262923. Online ahead of print. doi: 10.1001/jamapsychiatry.2026.2923 (PMC13647186; doi:10.1001/jamapsychiatry.2026.2923)
Supplement: Supplementary file 2 [file jamapsychiatry-e262923-s002.pdf]

Data Sharing Statement

Bergson. Characterizing Artificial Intelligence Psychosis in a Large Medical Setting. *JAMA Psychiatry*. Published October 07, 2026. doi:10.1001/jamapsychiatry.2026.2923

Data
Data available: No

Additional Information
Explanation for why data not available: Data for this study cannot be shared due to HIPAA concerns involving the distribution of personal health information.
